# Elevated serum soluble programmed cell death ligand 1 concentration as a potential marker for poor prognosis in small cell lung cancer patients with chemotherapy

**DOI:** 10.1186/s12931-018-0885-x

**Published:** 2018-10-05

**Authors:** Jianjun Jin, Jiming Si, Yuanhua Liu, Huanqin Wang, Ran Ni, Jing Wang

**Affiliations:** Department of Respiratory and Critical Medicine, the First Affiliated Hospital of Zhengzhou University, No. 1, Jianshe East Road, Zhengzhou, 450052 Henan Province China

**Keywords:** Chemotherapy, Prognosis, Programmed cell death ligand 1, Response, Small cell lung carcinoma

## Abstract

**Background:**

Potential relationship between serum soluble programmed cell death ligand 1 and prognosis of small cell lung cancer is not well explored. The aim of the study was to reveal the prognostic significance of serum soluble programmed cell death ligand 1 in patients with small cell lung cancer.

**Methods:**

A total of 250 small cell lung cancer patients and 250 controls were included. Research information was obtained from their medical records. Blood samples were collected on admission. Serum concentration of programmed cell death ligand 1 was measured using Enzyme-Linked Immunosorbent Assay. The patients underwent cisplatin-etoposide chemotherapy with a maximum of six cycles. Subsequently, they were followed-up for 12 months, and therapeutic response and cancer death were recorded.

**Results:**

Serum concentration of programmed cell death ligand 1 was higher in the patients than in the controls on admission (*P* < 0.001). After chemotherapy, 112 patients had no response to this therapy. In the 12-month follow up period, 118 patients died due to this cancer. Multivariate Cox regression model revealed that the higher serum concentration of programmed cell death ligand 1 on admission was associated with the higher risk of no response to chemotherapy or cancer caused death (HR: 1.40, 95% CI: 1.05 ~ 1.87; HR: 1.43, 95% CI: 1.08 ~ 1.87).

**Conclusion:**

Elevated serum concentration of soluble programmed cell death ligand 1 might be an independent risk factor for non-response to chemotherapy and cancer caused death in small cell lung cancer patients.

## Background

Lung cancer is the most common cause of cancer death in men and the second common cause in women around the world [1, 2]. There are two major histological lung cancers: non-small cell lung cancer (NSCLC) and small cell lung cancer (SCLC). SCLC accounts for about 15% of the cases [2]. Patients with SCLC primarily received systemic chemotherapy [3]. Cisplatin, etoposide, carboplatin, gemcitabine, paclitaxel, vinorelbine, topotecan, irinotecan and their combinations are commonly used in these patients [4, 5].

Programmed death 1 receptor (PD-1) and its ligand programmed death ligand 1 (PD-L1) are ectopically up-regulated in tumor tissue [6]. They attenuate the activation of T-cells and inhibit the anti-tumor immune response [7]. Due to these characteristics, PD-1 and PD-L1 are regarded as a novel target for immunotherapy, and their inhibitors have been adopted in NSCLC patients [8–10].

Several studies suggested that tissue expressions of PD-1 and PD-L1 were associated with survival in patients with SCLC or NSCLC [11, 12]. Furthermore, Okuma et al. in their study suggested that high serum level of soluble PD-L1 was prognostic for reduced survival in advanced lung cancer, but this study only included seven SCLC patients [13]. Therefore, prognostic significance of soluble PD-L1 in SCLC patients has not been well investigated.

A potential relationship between PD-L1 expression and chemotherapeutic response in NSCLC patients has been reported. Zhang et al. suggested that high expression of PD-L1 after cisplatin-based neo-adjuvant chemotherapy could be an indication of therapeutic resistance and poor prognosis in patients with NSCLC [14]. On the contrary, Ishii et al. reported that PD-L1 expression was associated with high immunoscore, and the highest immunoscore tended to have a favorable disease-free survival in NSCLC [15]. However, studies focusing on this relationship in SCLC patients are lacking.

This study aimed to evaluate the prognostic significance of soluble PD-L1 in SCLC, and explore the relationship between soluble PD-L1 and chemotherapeutic response in this disease.

## Methods

The study was approved by the ethics committee of the First Affiliated Hospital of Zhengzhou University.

### Subjects

A total of 250 patients with SCLC were continuously included from Department of Respiratory and Critical Medicine, the First Affiliated Hospital of Zhengzhou University between January 2010 and December 2016. Adequate histological samples with abundant tumor cells from these patients were available, and all these patients were diagnosed by pathology. Patients who did not have enough histological samples were excluded from this study. And, 250 health volunteers were randomly selected from medical examination center, the First Affiliated Hospital of Zhengzhou University in the same period, and served as controls. The patients did not receive any type of anti-tumor therapy before. The subjects did not have acute myocardial infarction, unstable angina, acute cerebral infarction/hemorrhage, severe infection, autoimmune disease or other kind of cancer. The patients and controls agreed to take part in this study, and signed the written informed consents.

### Data collection

Demographic and clinical information were obtained from their medical records. Tumor tissue expressions of epidermal growth factor receptor (EGFR) and kirsten rat sarcoma viral oncogene (KRAS) were obtained from their pathological records.

### Enzyme-linked immunosorbent assay

Blood samples were collected from the patients and controls on admission, and were immediately centrifuged at 1000 rpm for 12 min. Serum samples were stored at − 70 °C for following determination.

Serum concentration of PD-L1 was measured using a Human/Cynomolgus Monkey PD-L1/B7-H1 Quantikine Enzyme-Linked Immunosorbent Assay (ELISA) Kit (R&D Systems, Minneapolis, MN, USA) according to the manufacturer’s instruction. Sensitivity was 4.52 pg/ml, and assay range was 25.0–1600 pg/ml. Each sample was measured twice, and the mean was reported.

### Immunohistochemistry

Of the 250 SCLC patients, 98 provided available tumor tissue samples, which were stored at − 196 °C (liquid nitrogen) for further measurement.

Mature and reliable immunohistochemical staining technique was adopted to detect the PD-L1 expression in tumor tissue, and its steps were briefly introduced. First, the tissue samples were fixed in formalin (10%), embedded in paraffin and cut into 4 μm slice. Second, the slices were dewaxed in xylene, rehydrated using graded ethanol and rinsed in phosphate buffer saline. Third, antigen retrieval was performed in citrate buffer (0.01 mol/l, pH 6.0). Fourth, the slices were heated using microwave and then cooled at room temperature for 30 min. Fifth, the slices were infiltrated in hydrogen peroxide (3%) at room temperature for 20 min and incubated in goat serum at 37 °C for 40 min. Sixth, the slices were incubated with primary anti- PD-L1 monoclonal antibodies (Catalog Number: 66248–1-Ig; Proteintech, Wuhan, Hubei, China; 1:500 Dilution) at 4 °C overnight. Seventh, after washing in phosphate buffer saline, the slices were incubated with goat anti-mouse IgG (H + L), biotin conjugate secondary antibodies (Catalog Number: SA00004–1; Proteintech, Wuhan, Hubei, China; 1:500 Dilution) at 37 °C for 30 min. Eighth, the slices were exposed to 3, 3′-diaminobenzidine and counterstained in hematoxylin. Percentage of the positive staining SCLC cells was reported. If more than 5% of the SCLC cells in one tissue slice exhibited positive staining, it was considered as positive result [16].

### Definition

Tumor staging was determined by the veterans administration lung cancer group (VALG) staging system [17]. Limited disease (LD) was defined as an area that was tolerably treated by one radiotherapy area, but excluded cancers with pleural or pericardial effusions. All other SCLCs were regarded as extensive disease (ED). Performance status was measured according to the eastern cooperative oncology group (ECOG) score [18].

Smoker was defined as a subject who had at least one cigarette per week for six months or more in his or her life. Other subjects were defined as non-smokers.

### Therapy

The patients underwent chemotherapy with a maximum of six cycles. Their chemotherapy regimen was cisplatin and etoposide. Therapeutic response was defined according to the Response Evaluation Criteria in Solid Tumors (RECIST) [19]. There were four categories: complete response (CR), partial response (PR), stable disease (SD) and progressive disease (PD). In this study, CR and PR were grouped as response, and SD and PD were combined as non-response.

### Follow up

All the patients were followed-up for 12 months by telephone. The process was conducted by a well-trained investigator. Major endpoint was SCLC-caused death.

### Statistical analysis

Continuous variable was expressed as mean ± standard deviation (SD), and categorical variable was showed as frequency and constituent ratio. Difference of continuous variables was analyzed using independent sample t test, and difference of categorical variables was detected using chi-square test. A two-sided *P* < 0.05 was regarded to be statistically significant. A cutoff value for serum concentration of PD-L1 was calculated by receiver operating characteristic curve analysis. Relationship between serum concentration of PD-L1 and prognosis of SCLC patients was analyzed using multivariate Cox regression analysis. Hazard ratio (HR) and 95% confidence interval (CI) were reported. If a 95%CI included value one, it was considered statistically significant. All statistical analyses were conducted using SPSS 19.0 (SPSS Inc., Chicago, IL, USA).

## Results

As shown in Table 1, there were 250 SCLC patients and 250 health controls in the study. There was no difference in age and gender between these two groups (*P* = 0.381, *P* = 0.143). Smokers were more common in the SCLC patients than in the health controls (*P* = 0.012). Serum concentration of PD-L1 was higher in the SCLC patients than in the controls (*P* < 0.001).

**Table 1:** Baseline characteristics of the patients and controls

	SCLC ^a^	Control	*P* value
Total (*n*)	250	250	–
Age (*n*)	64.5 ± 5.8	65.0 ± 5.8	0.381
Gender (*n*)			
Female	53	67	0.143
Male	197	183	
Smoking history (*n*)			
Non-smoker	107	135	0.012
Smoker	143	115	
Performance status			
0–1	218	–	–
2	32	–	–
Tumor grade (*n*)			
High-middle	193	–	–
Low	57	–	–
Tumor stage (*n*)			
LD^a^	143	–	–
ED^a^	107	–	–
EGFR (*n*)^a^			
Negative	202	–	–
Positive	48	–	–
KRAS (*n*)^a^			
Negative	234	–	–
Positive	16	–	–
Serum PD-L1 (ng/ml)^a^	7.0 ± 3.0	1.2 ± 0.6	<0.001

^a^*SCLC* Small cell lung cancer, *LD* Limited disease, *ED* Extensive disease, *EGFR* Epidermal growth factor receptor, *KRAS* Kirsten rat sarcoma viral oncogene, *PD-L1* Programmed death ligand 1

As shown in Table 2, a total of 138 patients had a response and 112 patients had no response to chemotherapy. Age, gender, smoking history, performance status, tumor grade and tumor stage were equivalent between the patients with a response and the patients without a response to chemotherapy (*P* = 0.472, *P* = 0.817, *P* = 0.127, *P* = 0.076, *P* = 0.169, *P* = 0.312). Serum concentration of PD-L1, tissue expressions of EGFR and KRAS were higher in the patients without a response than in the patients with a response to chemotherapy (*P* = 0.008, *P* = 0.016, *P* = 0.046).

**Table 2:** Baseline characteristics of the non-response and response patients

	Non-response	Response	*P* value
Total (*n*)	112	138	–
Age (*n*)	64.2 ± 6.2	64.7 ± 5.5	0.472
Gender (*n*)			
Female	23	30	0.817
Male	89	108	
Smoking history (*n*)			
Non-smoker	42	65	0.127
Smoker	70	73	
Performance status			
0–1	93	125	0.076
2	19	13	
Tumor grade (*n*)			
High-middle	91	102	0.169
Low	21	36	
Tumor stage (*n*)			
LD^a^	68	75	0.312
ED^a^	44	63	
EGFR (*n*)^a^			
Negative	83	119	0.016
Positive	29	19	
KRAS (*n*)^a^			
Negative	101	133	0.046
Positive	11	5	
Serum PD-L1 (ng/ml)^a^	7.6 ± 2.5	6.6 ± 3.2	0.008

^a^*LD* Limited disease, *ED* Extensive disease, *EGFR* Epidermal growth factor receptor, *KRAS* Kirsten rat sarcoma viral oncogene, *PD-L1* Programmed death ligand 1

As shown in Tables 3, 118 patients died due to SCLC in the follow up period, and the remaining 132 patients were still living at the end of the follow up. There was no difference in age, gender, smoking history, performance status, tissue expressions of EGFR and KRAS between the dead patients and the living patients (*P* = 0.573, *P* = 0.532, *P* = 0.370, *P* = 0.063, *P* = 0.086, *P* = 0.074). More dead patients had low-grade or ED-stage tumors (*P* = 0.014, *P* = 0.007). More dead patients had no response to chemotherapy (*P* < 0.001). Serum concentration of PD-L1 was higher in the dead patients than in the living patients (*P* = 0.003).

**Table 3:** Baseline characteristics of the cancer-dead and survival patients

	Cancer death	Survival	P value
Total (*n*)	118	132	–
Age (*n*)	64.2 ± 5.5	64.7 ± 6.1	0.573
Gender (*n*)			
Female	23	30	0.532
Male	95	102	
Smoking history (*n*)			
Non-smoker	47	60	0.370
Smoker	71	72	
Performance status			
0–1	98	120	0.063
2	20	12	
Tumor grade (*n*)			
High-middle	83	110	0.014
Low	35	22	
Tumor stage (*n*)			
LD^a^	57	86	0.007
ED^a^	61	46	
EGFR (n)^a^			
Negative	90	112	0.086
Positive	28	20	
KRAS (*n*)^a^			
Negative	107	127	0.074
Positive	11	5	
Chemotherapeutic response (*n*)			
Response	12	126	<0.001
Non-response	106	6	
Serum PD-L1 (ng/ml)^a^	7.6 ± 3.1	6.5 ± 2.7	0.003

^a^*LD* Limited disease, *ED* Extensive disease, *EGFR* Epidermal growth factor receptor, *KRAS* Kirsten rat sarcoma viral oncogene, *PD-L1* Programmed death ligand 1

**Fig. 1:**
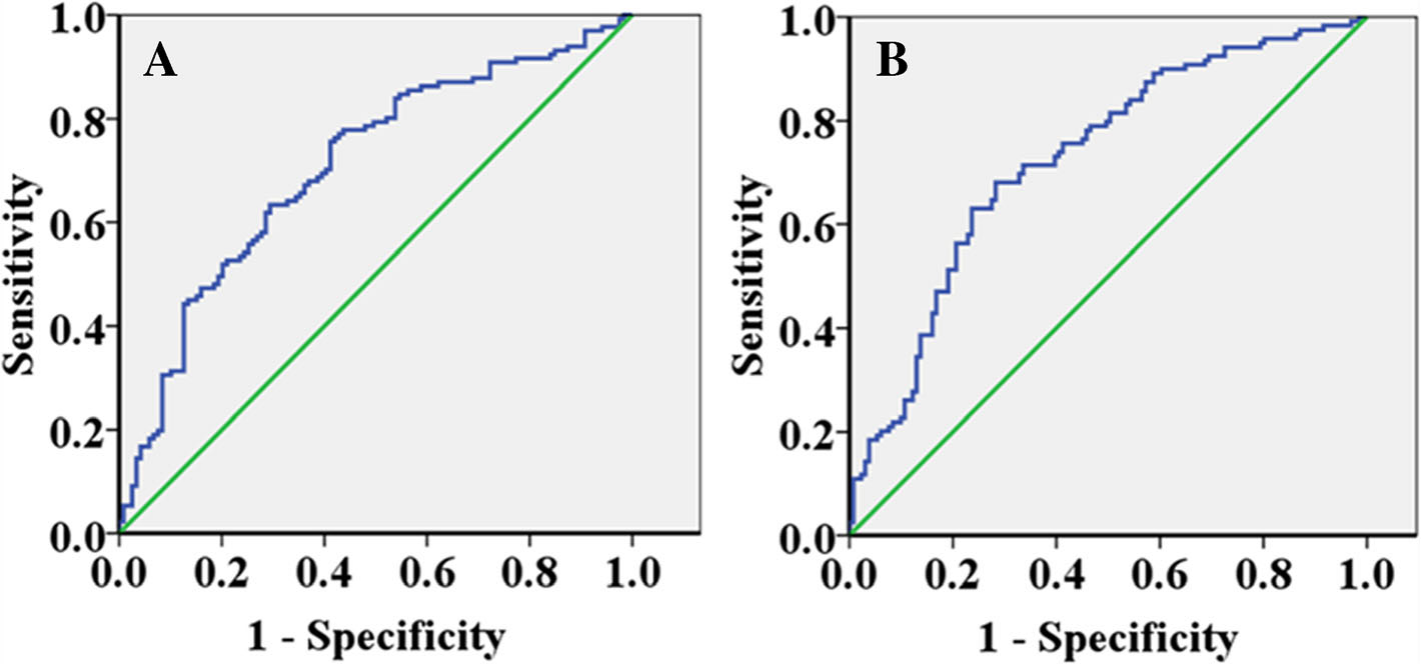
Receiver operating characteristic curve analysis of serum soluble programmed cell death ligand 1 in the patients according to the prognosis. **a**. A cutoff point for serum concentration of soluble programmed cell death ligand 1 was 7.0 ng/ml in predicting the risk of chemotherapeutic non-response (Sensitivity = 68.7%, Specificity = 69.8%, Area under the curve = 0.707, *P* < 0.001). **b**: A cutoff point for serum concentration of soluble programmed cell death ligand 1 was 7.1 ng/ml in predicting the risk of cancer death (Sensitivity = 73.9%, Specificity = 71.3%, Area under the curve = 0.726, *P* < 0.001)

A cutoff point for serum concentration of soluble PD-L1 was 7.0 ng/ml in predicting the risk of chemotherapeutic non-response (Sensitivity = 68.7%, Specificity = 69.8%, Area under the curve = 0.707, *P* < 0.001) (Fig. 1a). A cutoff point for serum concentration of soluble PD-L1 was 7.1 ng/ml in predicting the risk of cancer death (Sensitivity = 73.9%, Specificity = 71.3%, Area under the curve = 0.726, *P* < 0.001) (Fig. 1b).

**Fig. 2:**
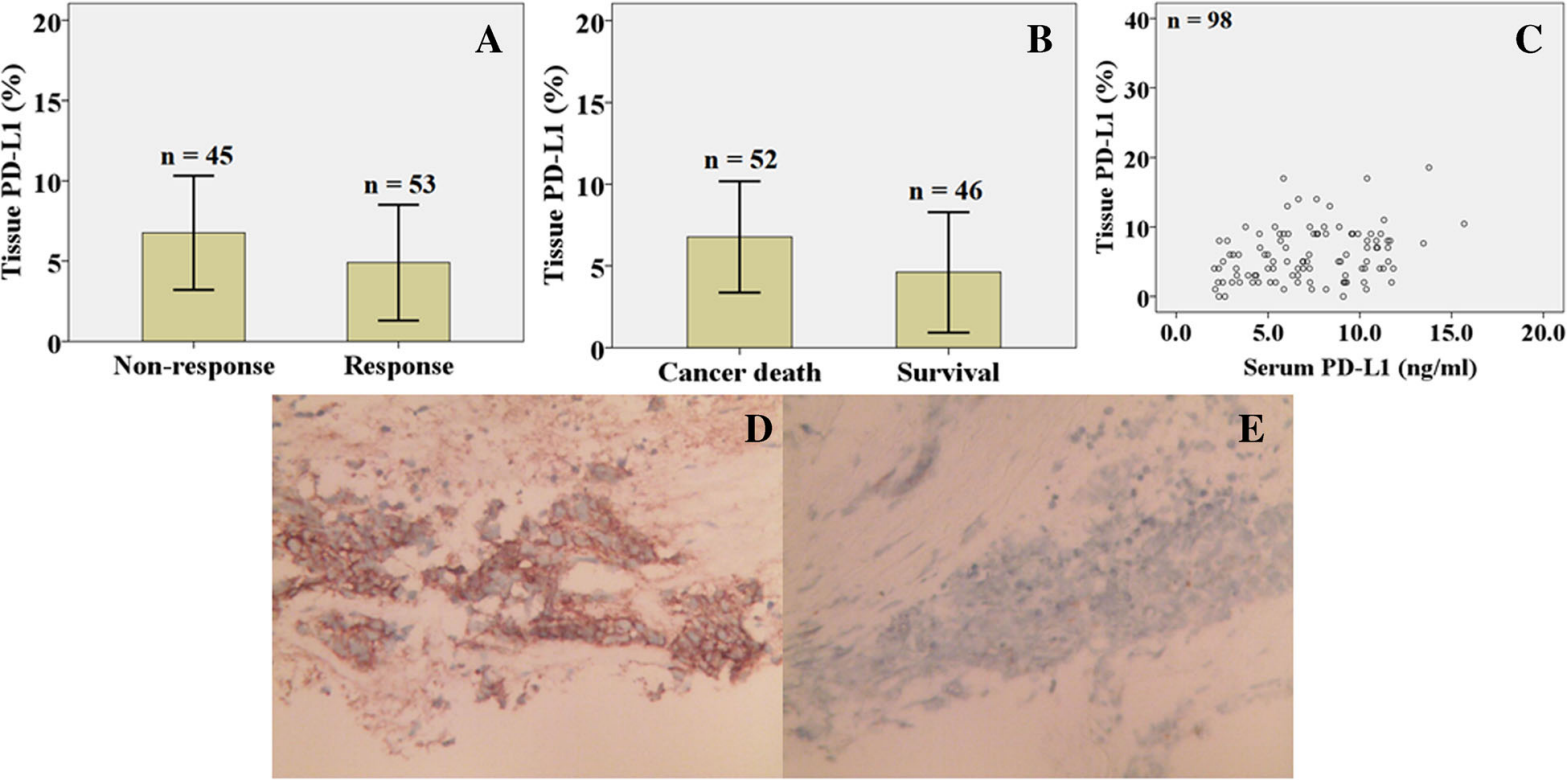
Tissue expression of soluble programmed cell death ligand 1 in patients with small cell lung cancer patients. **a**: Tissue expression of programmed cell death ligand 1 was higher in the patients without a response to chemotherapy than in the patients with a response to chemotherapy (6.8% ± 3.6%, 4.9% ± 3.6%, *P* = 0.012). **b**: Tissue expression of programmed cell death ligand 1 was higher in the dead patients than in the living patients (6.8% ± 3.4%, 4.6% ± 3.7%, *P* = 0.003). **c**: There is a linear relationship between serum level of soluble programmed cell death ligand 1 and tissue expression of soluble programmed cell death ligand 1 (*r* = 0.214, *P* = 0.034). **d**: Positively immunohistochemical staining of programmed death ligand 1 in patients with small cell lung cancer patients (400×). **e**: Negatively immunohistochemical staining of programmed death ligand 1 in patients with small cell lung cancer patients (400×)

Tissue expression of PD-L1 was higher in the patients without a response than in the patients with a response to chemotherapy (6.8% ± 3.6%, 4.9% ± 3.6%, *P* = 0.012) (Fig. 2a). Tissue expression of PD-L1 was higher in the dead patients than in the living patients (6.8% ± 3.4%, 4.6% ± 3.7%, *P* = 0.003) (Fig. 2b). In Fig. 2c, there is a linear relationship between serum level of soluble PD-L1 and tissue expression of PD-L1 (*r* = 0.214, *P* = 0.034). Figure 2d and e showed positively and negatively immunohistochemical staining of PD-L1 in SCLC patients, respectively.

According to the cutoff values of the serum PD-L1 level, the patients were divided into several subgroups for following analysis. As shown in Tables 4 and 5, the higher serum concentration of soluble PD-L1 was associated with the higher risk of no response to chemotherapy or SCLC caused death (HR: 1.40, 95% CI: 1.05 ~ 1.87; HR: 1.43, 95% CI: 1.08 ~ 1.87). The positive expression of PD-L1 in tissue was also associated with the higher risk of no response to chemotherapy or SCLC caused death (HR: 1.76, 95% CI: 1.13 ~ 2.78; HR: 1.86, 95% CI: 1.24 ~ 2.76). In addition, the higher expressions of EGFR and KRAS were related to the higher risk of no response to chemotherapy (HR: 1.45, 95% CI: 1.09 ~ 1.93; HR: 1.57, 95% CI: 1.09 ~ 2.27). Low-grade and ED-stage tumors were associated with the higher risk of cancer death (HR: 1.41, 95% CI: 1.08 ~ 1.85; HR: 1.42, 95% CI: 1.09 ~ 1.84).

**Table 4:** Relationship between several markers and chemotherapeutic response

	Non-response (*n*)	Total (*n*)	Multivariate HR^a^ (95% CI)^a,b^	Multivariate HR (95% CI)^c^
Total	112	250	–	–
Tumor grade				
High-middle	91	193	Reference	Reference
Low	21	57	0.78 (0.54 ~ 1.13)	0.76 (0.53 ~ 1.11)
Tumor stage				
LD^a^	68	143	Reference	Reference
ED^a^	44	107	0.86 (0.65 ~ 1.15)	0.84 (0.64 ~ 1.13)
EGFR^a^				
Negative	83	202	Reference	Reference
Positive	29	48	1.47 (1.11 ~ 1.95)	1.45 (1.09 ~ 1.93)
KRAS^a^				
Negative	101	234	Reference	Reference
Positive	11	16	1.59 (1.11 ~ 2.29)	1.57 (1.09 ~ 2.27)
Serum PD-L1^a^				
<7.0 ng/ml	45	122	Reference	Reference
≥ 7.0 ng/ml	67	128	1.42 (1.07 ~ 1.89)	1.40 (1.05 ~ 1.87)
Tissue PD-L1				
Negative	17	50	Reference	Reference
Positive	28	48	1.75 (1.12 ~ 2.77)	1.76 (1.13 ~ 2.78)

^a^LD: Limited disease; ED: Extensive disease; EGFR: Epidermal growth factor receptor; KRAS: Kirsten rat sarcoma viral oncogene; PD-L1: Programmed death ligand 1; HR: Hazard ratio; CI: Confidence interval

^b^The model was adjusted by age and gender

^c^The model was adjusted by age, gender, smoking history, performance status, tumor grade, tumor stage, tissue expressions of epidermal growth factor receptor and kirsten rat sarcoma viral oncogene

**Table 5:** Relationship between several markers and cancer caused death

	Cancer death (*n*)	Total (*n*)	Multivariate HR^a^ (95% CI)^a, b^	Multivariate HR (95% CI) ^c^
Tumor grade				
High-middle	83	193	Reference	Reference
Low	35	57	1.43 (1.10 ~ 1.86)	1.41 (1.08 ~ 1.85)
Tumor stage				
LD^a^	57	143	Reference	Reference
ED^a^	61	107	1.43 (1.10 ~ 1.85)	1.42 (1.09 ~ 1.84)
EGFR^a^				
Negative	90	202	Reference	Reference
Positive	28	48	1.31 (0.99 ~ 1.74)	1.30 (0.98 ~ 1.73)
KRAS^a^				
Negative	108	234	Reference	Reference
Positive	10	16	1.35 (0.90 ~ 2.03)	1.33 (0.88 ~ 2.01)
Serum PD-L1^a^				
<7.1 ng/ml	48	124	Reference	Reference
≥ 7.1 ng/ml	70	126	1.44 (1.09 ~ 1.88)	1.43 (1.08 ~ 1.87)
Tissue PD-L1				
Negative	19	50	Reference	Reference
Positive	33	48	1.85 (1.24 ~ 2.75)	1.86 (1.24 ~ 2.76)

^a^*LD* Limited disease, *ED* Extensive disease, *EGFR* Epidermal growth factor receptor, *KRAS* Kirsten rat sarcoma viral oncogene, *PD-L1* Programmed death ligand 1, *HR* Hazard ratio, *CI* Confidence interval

^b^The model was adjusted by age and gender

^c^The model was adjusted by age, gender, smoking history, performance status, tumor grade, tumor stage, tissue expressions of epidermal growth factor receptor and kirsten rat sarcoma viral oncogene

## Discussion

To our knowledge, this is the first published article focusing on the relationship between serum concentration of soluble PD-L1 and prognosis of SCLC patients after chemotherapy. There were 250 patients with confirmed SCLC and 250 health controls in the study. We discovered that serum concentration of soluble PD-L1 significantly elevated in the SCLC patients, which was consistent with a previous study involving several types of advanced lung cancers [13].

A previous study suggested that soluble PD-L1 originated from PD-L1 on the surface of tumor cells by a disengagement mechanism, and serum concentration of soluble PD-L1 should be related to the tumor burden [20]. In the study, we confirmed the relationship between the serum level of soluble PD-L1 and the expression of PD-L1 in tissue. However, this did not mean that the soluble PD-L1 was also associated with the tumor burden, because the expression level of PD-L1 per unit tumor volume was diverse.

Serum PD-L1 was not a specific tumor marker, but an inflammatory and immunoregulatory marker in human [21]. Soluble PD-1 and its receptor PD-1 could be produced by peripheral immunological cells, and represented an unanticipated contributing factor to immune homeostasis [22]. Greisen et al. reported that increased concentration of soluble PD-1 was associated with disease activity and radiographic progression in rheumatoid arthritis [23]. Wu et al. and Shi et al. suggested that soluble PD-1 also contributed to the aberrant activation of T cells and the development of diseases in aplastic anemia and diabetic atherosclerotic complications, respectively [24, 25]. So, an inflammatory lesion in tumor as well as an extra-tumor inflammatory lesion could elevate the concentration of soluble PD-L1. In SCLC patients without other inflammation and immune related diseases, serum concentration of soluble PD-L1 indicated the extents of anti-tumor T cell response and immune suppression induced by PD-1 and PD-L1.

In the study, we reported a 40% increased risk of non-response to chemotherapy in the patients with relatively higher concentration of soluble PD-L1, which was consistent with one previous study by Zhang et al. [14] and was contrary to another study by Ishii et al. [15]. Both these studies included NSCLC patients and measured the tissue expression of PD-L1, but not the serum concentration of PD-L1.

Some studies had revealed the effect of PD-L1 on chemoresistance in other type of cancer. Black et al. suggested that the activation of PD-1/PD-L1 immune checkpoint conferred breast cancer cell chemoresistance associated with increased metastasis [26]. Ishibashi et al. found that the interaction between PD-1 and PD-L1 not only inhibited tumor-specific cytotoxic T lymphocytes, but also induced drug resistance in myeloma cells [27]. Tamura et al. reported that the bone marrow microenvironment up-regulated PD-1 expression on myeloma cells, which linked to aggressive myeloma-cell characteristics and insensitivity to anti-myeloma chemotherapy [28].

Tumor infiltrating lymphocyte (TIL) was regarded to be a protective factor for cancer, and it improved survival in NSCLC patients [29]. Zhang et al. suggested that the higher TIL expression rate was usually found in chemosensitive samples in NSCLC [14]. Potential association between PD-L1 and TILs was also confirmed in lung cancer patients [30]. Furthermore, miR-197/CKS1B/STAT3-mediated network regarded the tumor expression of PD-L1 as a biomarker of this cascade, and the biological interaction between PD-L1 and chemoresistance occurred through this microRNA regulatory cascade [31]. Therefore, the effect of PD-L1 on chemoresistance might be involved in the inhibition of TILs and the dysregulation of miR-197/CKS1B/STAT3-mediated cascade. These findings partly explained the effect of PD-L1 on chemotherapeutic response.

Furthermore, our study revealed a relationship between soluble PD-L1 and prognosis of SCLC patients, and the patients with higher concentration of soluble PD-L1 showed a 40% increased risk of cancer caused death in the 12-month follow up period. This result was consistent with a previous study exploring the prognostic significance of soluble PD-L1 in advanced lung cancer [13]. In the study, most of the dead patients had no response to chemotherapy. Therefore, prognostic significance of soluble PD-L1 might be related to the effect of this ligand on chemotherapeutic response.

Prognostic significance of soluble PD-L1 in other type of cancer had also been explored. Zheng et al. suggested that circulating PD-L1 expression was significantly correlated with differentiation and lymph node metastasis in total advanced gastric cancer patients [32]. Ha et al. reported that advanced biliary tract cancer patients with high soluble PD-L1 showed worse overall survival than patients with low soluble PD-L1, and high soluble PD-L1 was an independent poor prognostic factor in multivariate analysis [33]. Wang et al. revealed that overall response rate to treatment in multiple myeloma patients was higher in low soluble PD-L1 patients than in high soluble PD-L1 patients, and higher soluble PD-L1 level was an independent prognostic factor for shorter 3-year progression free survival [34]. Finkelmeier et al. discovered that soluble PD-L1 level positively correlated with the stage of cirrhosis and with stage of HCC, and patients with high serum PD-L1 concentration had an increased mortality risk, while very low PD-L1 level seemed to come along with better prognosis [35]. Rossille et al. suggested that diffuse large B-cell lymphoma patients with elevated soluble PD-L1 experienced a poorer prognosis with a 3-year overall survival of 76% versus 89% [36]. A meta-analysis from Zhu et al. indicated that high PD-L1 expression was likely to be a negative factor for patients with sarcomas and that it predicted worse survival outcomes [37].

PD-1 and PD-L1 had become a target for anti-tumor therapy. According to this understanding, a novel targeted drug nivolumab had been produced, which was an IgG4 monoclonal antibody against PD-1. It exerted its biological function through inhibiting the PD-1/PD-L1 signal pathway and allowing the immune system to scavenge the tumor cells [38]. Around the world, nivolumab had been used in the treatment of metastatic melanoma, squamous non-small cell lung cancer and renal cell carcinoma [39–41]. Potential therapeutical effect of the drug on SCLC had also been explored. A multicentre, open-label, phase 1/2 trial at 23 hospitals in six countries (CheckMate 032) reported that nivolumab monotherapy and nivolumab plus ipilimumab had an anti-tumor activity with durable responses and manageable safety profiles in previously treated patients with SCLC [42]. Furthermore, Hellmann et al. in their study reported that the efficacy of nivolumab monotherapy and nivolumab plus ipilimumab was enhanced in SCLC patients with high tumor mutational burden [43]. This promising finding required further confirmation in phase 3 randomized controlled trials in SCLC.

## Conclusion

In conclusion, elevated serum concentration of soluble PD-L1 might be an independent risk factor for non-response to chemotherapy and cancer caused death in SCLC patients. Further studies should be conducted to explore the mechanisms involved.

## Abbreviations

CI: Confidence interval
CR: Complete response
ECOG: Eastern cooperative oncology group
ED: Extensive disease
EGFR: Epidermal growth factor receptor
ELISA: Enzyme Linked Immunosorbent Assay
HR: Hazard ratio
KRAS: Kirsten rat sarcoma viral oncogene
LD: Limited disease
NSCLC: Non-small cell lung cancer
PD: Progressive disease
PD-1: Programmed death 1 receptor
PD-L1: Programmed death ligand 1
PR: Partial response
RECIST: Response Evaluation Criteria in Solid Tumors
SCLC: Small cell lung cancer
SD: Stable disease
SD: Standard deviation
TIL: Tumor infiltrating lymphocyte
VALG: Veterans administration lung cancer group
